# Wild Hops in Breadmaking Among Bulgarians: From History to Modern Perspectives and Future Potentials

**DOI:** 10.3390/foods14101767

**Published:** 2025-05-16

**Authors:** Anely Nedelcheva, Dauro Mattia Zocchi, Naji Sulaiman, Renata Sõukand, Andrea Pieroni, Antonella Pasqualone

**Affiliations:** 1 Department of Organic Chemistry and Pharmacognosy, Faculty of Chemistry and Pharmacy, Sofia University “St. Kliment Ohridski”, 1 James Bourchier Blvd., 1164 Sofia, Bulgaria; ohan@chem.uni-sofia.bg; 2 Department of Foreign Languages, Literatures and Cultures, University of Bergamo, Piazza Rosate 2, 24129 Bergamo, Italy; dauromattia.zocchi@unibg.it; 3 University of Gastronomic Sciences, Piazza Vittorio Emanuele II, 9, 12042 Pollenzo, Italy; n.sulaiman@unisg.it (N.S.); a.pieroni@unisg.it (A.P.); 4 Department of Environmental Sciences, Informatics and Statistics, Ca’ Foscari University of Venice, Via Torino 155, 30174 Venezia, Italy; renata.soukand@unive.it; 5 Department of Medical Analysis, Tishk International University, Erbil 44001, Iraq; 6 Department of Soil, Plant and Food Science (DISSPA), University of Bari “Aldo Moro”, Via Amendola, 165/a, 70126 Bari, Italy

**Keywords:** *Humulus lupulus* L., Balkans, food heritage, food processing, *kvass*, phytochemicals, sourdough, traditional bread, wild plants

## Abstract

*Humulus lupulus* L. (common hop) is a herbaceous plant whose female inflorescences, commonly called hop “cones”, are traditionally used in Bulgaria to prepare sourdough starters or “*kvass*”. Drawing from a review of historical and linguistic sources and ethnographic information collected by the authors, this study aims to define the traditional preparation of bread with hop sourdough, starting from the preparation of the hop cone decoction. Archival materials and early cookbooks attest to a rich tradition where hop-infused bread was valued for its distinctive flavor and preservative qualities. Fieldwork conducted in Bulgaria and among Bulgarian diasporas in Moldova provided insights into the continuity of these practices, underscoring the persistence of these traditional preparations despite modern industrial pressures. Ethnographic interviews and participant observations highlighted the ritualistic preparation of hop *kvass* and its role in community identity. The effect of hops on dough’s rheological properties and the quality features of bread were also reviewed. An increase in dough stability and resistance to elongation were generally reported, with a reduction in bread volume and porosity, especially with hop sourdough levels above 30%, but the incorporation of bioactive molecules was responsible for antioxidant, antimicrobial, and flavoring properties. Possible prospects for using hops in the food industry, based on the biological properties of this resource-rich plant, are outlined with a multidisciplinary approach.

## 1. Introduction

*Humulus lupulus* L. (common hop) is a herbaceous perennial liana, one of three *Humulus* species in the family *Cannabaceae* [1]. The female inflorescences of the hop plant, or strobili, commonly referred to as hop “cones”, are essential for the characteristic bitter taste and flavor of beer. Still, due to their secondary metabolites, such as phenolic compounds, essential oils, and resins, they also exhibit significant health effects, for which they have been traditionally used in folk medicine [2]. These metabolites also show antibacterial and antifungal activity that help prevent beer spoilage.

Besides its well-known use in beer brewing, *H. lupulus* has been traditionally used for various purposes since ancient times [3]. Previous studies refer to ethnobotanical uses such as dying hair, fabric and paper production, cattle fodder and, in folk medicine, against leprosy, toothache, fever, gastric problems, and anxiety [1,2,3]. Sõukand et al. [4] reported that *H. lupulus* is among the top quoted species in the Balkan region, where it is associated with old traditions. Traditional food, deeply rooted in the local environment, plays a significant role in the culture and economy of local communities, but many of the practices and craft skills related to these foods are often based on the knowledge of elders and are in danger of being forgotten.

Bread is a regularly consumed product that can be enriched with functional ingredients, serving as a carrier for biologically active compounds. Plant-derived ingredients, including hop extracts, are among the most frequently considered for conferring functional properties to bread [5]. Nedelcheva and Dogan [6], in their ethnobotanical review on the use of plants employed in the preparation of sourdough starters for breadmaking in the Balkans, noted the traditional use of a decoction made from wild hop cones in Bulgaria. Similar use of hop cones exists among Bulgarian communities living in the southern part of Moldova, especially among the elderly women, as discovered in a recent ethnobotanical survey [7]. The latter, however, was an extensive study that recorded the food use of sixty-six wild and semi-domesticated plants and fungi making it impossible to fully explore the details of the recipes and processing steps.

In addition to the need to keep traditions alive as a sign of local identity and culture, old traditional practices related to food preparation are attractive today because they can still meet, in an original or evolved form, modern consumers’ expectations of nutritious, healthy, and sustainable food products.

Based on these premises, the aim of this study was twofold: (i) to keep the knowledge about the use of wild hops in breadmaking alive by recording, in detail, the practices among Bulgarians and the Bulgarian diaspora in Moldova through ethnobiological and ethnographic fieldwork conducted by the authors and supported by historical and linguistic sources; (ii) to provide useful information for bakers and the food and beverage industry in general by reviewing the most recent technical and scientific studies on innovative food applications of hops.

Then, this multidisciplinary study provides an overview of the traditional preparation of bread with hop sourdough in Bulgaria and among the Bulgarian diaspora in Moldova, hitherto never studied in detail. In addition, this research defines the effect of hop addition on the quality properties of dough and bread and outlines possible prospects for using hops in the food industry based on the biological properties of this resource-rich plant, supporting innovation in the food sector.

## 2. Materials and Methods

The study adopted a multidisciplinary approach that involved several disciplines (history, ethnography, ethnobotany, languages, biology, food chemistry, and food technology) to comprehensively analyze and better understand the traditional knowledge of Bulgarians about the use of wild hops in breadmaking and the potential for the food industry. Analysis of the historical, linguistic, and technical–scientific literature was combined with ethnobiological and ethnographic fieldwork.

### 2.1. Literature Review

#### 2.1.1. Historical and Linguistic Sources

Methods and approaches for working with documentary and historical sources suggested by Bernard [8] and Rotem [9] were adopted. Following these approaches, the study was conducted in the following steps: formulating the objective, searching the existing literature, assessing the quality of primary studies, extracting data, summarizing previous investigations, and analyzing data. The objective was, through the collection of historical and literary information, to define the traditions related to bread and the use of hop sourdough among Bulgarians (presented in Section 3.1). The study was conducted in the period November 2023–January 2025 by accessing the archival materials (including photographs) from the Archives of the Institute of Ethnology and Folklore Studies with Ethnographic Museum at the Bulgarian Academy of Sciences (Sofia, Bulgaria) and from the following digital databases: Bulgarian ethnography (in Bulgarian) [10]; interactive culinary map of the Bulgarian language territory [11]; interactive dialect map of the Bulgarian language [12]; and the digital library of old printed books of the National Library “St. Cyril and Methodius” (Sofia, Bulgaria) [13].

#### 2.1.2. Technical–Scientific Sources

To define the effect of hop addition on the quality properties of dough and bread and to outline possible prospects of hop use in the food industry based on the biological properties of this plant, a comprehensive literature search was conducted by accessing the Scopus database over the period 2005–2024. Articles that showed the following search words in the title, abstract, and keywords were considered: “hop composition” (1 result), “hop extraction” (24 results), “hop” and “biological properties” (23 results), “hop” and “bread” (27 results), “hop” and “sourdough” (14 results), “hop” and “food application” (7 results), and “brewer’s spent hops” (7 results). After excluding duplicates and articles not relevant to the objectives of the study or with redundant content, a total number of 54 articles were reviewed. A traditional narrative review approach [14,15] was adopted to summarize the retrieved information, describing current knowledge (presented in Section 3.3) and identifying the need for new research.

### 2.2. Ethnobiological and Ethnographic Research

Fieldwork was conducted during two distinct periods, employing a shared methodological framework centered on food scouting. Utilizing a combination of ethnographic techniques alongside fundamental ethnobiological and ethnoecological approaches, the research aimed to gather baseline data on local food-related resources. These data serve as a foundation for investigating the integration of specific foodstuffs (and their associated practices and knowledge) within contemporary foodscapes, as well as for tracing their evolution across spatial and temporal dimensions [16].

In October 2022, fieldwork was conducted in four villages within the Autonomous Territorial Unit of Gagauzia and in Moldovan districts bordering the northern part of the Gagauz region—specifically, Aluatu, Sătuc, Taraclia, and Tvardița. These locations were selected based on an analysis of the socio-demographic characteristics and ethnic composition of the villages in the study area, using data from the 2014 Moldovan census released by the National Bureau of Statistics of the Republic of Moldova, which indicated a marked predominance of Bulgarian communities. Twenty-two face-to-face interviews were conducted, supplemented by participant observation and informal discussions with key informants, which enhanced the understanding of the food and ethnobotanical heritage of these communities. Informants were selected via convenience sampling with the criterion that they belonged to the Bulgarian ethnic community. Where possible, snowball sampling was also employed, allowing already identified participants to suggest additional informants from within the same community, thus facilitating access to individuals with relevant knowledge and enhancing the representativeness of the sample [7]. Interviews were conducted in various settings, including near participants’ homes, on public streets, and in workplaces such as gardens and food retail establishments. The sample predominantly comprised middle-aged and elderly people (9 men and 13 women; age range: 40–80 years), including farmers, food vendors, and housewives, who were identified as potential custodians of local knowledge. While most interviews were conducted individually, focus group interviews were occasionally employed to capture possible divergences in knowledge regarding food preparation and usage. The interviews focused on the preparation of homemade fermentation starters incorporating wild hops, the processing methods employed, the techniques applied in bread production, and the traditional consumption practices associated with these products. The interviews were conducted in Russian, and, on rare occasions, local interpreters were engaged to resolve language-related issues. Each interview lasted approximately 30 to 45 min.

In the period between October 2023 and January 2024, interviews were conducted with three local experts in Bulgaria regarding the processing steps for making hop kvass and homemade bread. The informants were a 70-year-old woman from the village of Lozen (Sofia region, Bulgaria), an 89-year-old man from the village of Emen (Veliko Tarnovo district, Bulgaria), and an 81-year-old woman from the village of Poletintsi (Kyustendil district, Bulgaria). The latter, in addition to giving a detailed description of the processing steps, performed a demonstration of the preparation of hop kvass and homemade bread, during which photographs were taken.

Before each interview, verbal informed consent was obtained from each interviewee, as recommended by the code of ethics of the International Society of Ethnobiology, and the rationale, aims, methods, and expected outputs of the project were explained to the interviewees in advance [17]. Participants were informed that their participation was entirely voluntary and that they could interrupt or withdraw from the interview at any time without any penalty.

In the fall of 2022 and 2023, a field study was conducted in the informal markets of six Moldovan centers—Chișinău, Orhei, Bălți, Călărași, Comrat, and Taraclia—through visits and informal conversations with 40 elderly women vendors that were selected via convenience sampling [18].

The interviews and the field notes were translated into English, transcribed and entered into NVivo version 12.5.0 (QSR International, Melbourne, Australia) [19], and then codes, concepts, and nodes were generated during the qualitative data analysis. All the data were organized and subsequently selected and condensed as tables and compared to highlight the different ingredients, steps and operations linked to the preparation of homemade fermentation starters incorporating wild hops, the ingredients and techniques applied in bread production, as well as the gastronomic uses of these products.

The results of ethnobiological and ethnographic fieldwork are presented in Section 3.2.

## 3. Results and Discussion

In the following section, the outcomes of the multidisciplinary investigation are presented and discussed. The analysis is organized into distinct subsections: the first examines bread traditions in the Balkans and among Bulgarians from a historical perspective, thereby establishing the contextual framework, followed by an exploration of the occurrence of hop sourdough in Bulgarian literary sources, which highlights its documented presence and cultural relevance. The subsequent subsections detail the traditional methodologies for preparing hop kvass and hopped bread and extend the investigation to include the preparation of dried hop kvass among the Bulgarian diaspora in Moldovan Bessarabia, as documented during the fieldwork conducted by the authors. The discussion then focuses on a critical assessment of the composition and biological properties of hop edible parts and evaluates the effect of hop addition on the quality properties of dough and bread. The section concludes with an analysis of further prospects for the exploitation of hops in food processing, thereby identifying potential avenues for future research.

### 3.1. Historical and Literary Background

#### 3.1.1. Bread Traditions in the Balkans and Among Bulgarians: Historical Perspectives

In Byzantine and Western European sources of the 11th–15th centuries, according to Bencheva’s analysis [20], medieval authors emphasized a difference in bread consumption between settled Christian populations and Balkan nomads (Pechenegs, Cumans, and Tartars). Nomads mainly consumed meat, dairy products, eggs from wild birds, nuts, and wild fruits and vegetables. Christians relied on agriculture and their diet included bread, which also had a religious connotation being used in the communion rite, in leavened form for Orthodox Christians and unleavened for Armenian and Western churches [21,22].

Travelogues to the Balkans written by French travelers between the 15th and 18th centuries [23,24,25] described the characteristics of Bulgarian bread as very poor, being a bread made with dark, whole-wheat flour, with a backward production technology that involved baking under hot ashes on an open hearth or in round clay vessels [26,27]. This method of baking still exists today in the region [28]. The appearance of the bread was described as unsightly and its taste as unpleasant [23,24,25], but Georgieva [26] makes an important point about the reliability of this information, commenting that it was primarily the impressions of a foreigner who was unfamiliar with the culture described and was not even aware of the ritual nature of bread [23,26].

**Figure 1:**
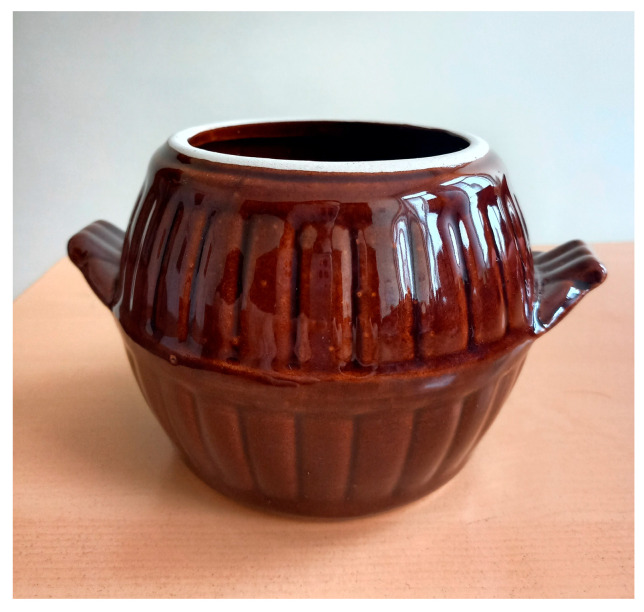
A typical clay pot, called a *kvasnik*, used to store the hop sourdough.

In the 15th to 18th centuries, a significant difference in practices related to the preparation and consumption of bread in rural *versus* urban areas has been reported [26,27,29]. Urban production was characterized by established standard technology, making it a well-paid craft. It was assumed that only part of the urban Bulgarian population could afford this type of bread, so home production was a common practice [27,29]. In villages, all stages of breadmaking were completed manually, and unleavened bread was more common [30]. Sourdough leavened bread was prepared for special rituals, following a long and laborious process. The Bulgarian term for “sourdough” is “*kvass*”—not to be confused with the fermented drink of the same name. The consistency of *kvass* or sourdough is thick, and the taste is slightly sour. In the past, *kvass* was present in every home: every woman prepared it for the family and carefully stored it in a special container intended only for this use, usually a clay pot (*kvasnik*) [квасник] [6] (Figure 1). Bulgarians used to start a new sourdough on Maundy Thursday (i.e., in Christianity, the holy day that falls on the Thursday before Easter), a time of year that symbolizes renewal [6].

Each tool used in the preparation of bread was made of wood, usually beech. Bread was kneaded and then fermented in wooden troughs called “*noshtvi*” [нoщви], made from a log [27,29]. This wooden trough was not washed after its use but only scraped. In this way, some dry residues of the fermented dough remained on the trough surface, which contributed their microbiota when flour and water were added to knead a new bread. A similar tradition was recorded in Ukraine, where the trough is called a “*netska*” or “*nochva*” [31].

Numerous folk beliefs surrounded sourdough, as it was not known how it could make bread rise. Under special circumstances related to folk beliefs, a non-proprietary or “foreign” sourdough starter was used, i.e., not prepared at home by the housewife; if one person in the house died, the *kvass* was exchanged for another, taken from another house [26]. It was also believed that a house without a *kvass* should not remain or that if the *kvass* was removed from the house, the wealth of the house would also be lost. The *kvass* was not given as a gift and the technology for its preparation was kept secret [26,29]. These customs, mostly typical of the past, are still observed today in Bulgaria [26,29].

#### 3.1.2. Occurrence of Hop Sourdough in Bulgarian Literary Sources

Because *H. lupulus* is a wild plant of the Bulgarian flora [32] and its cones are traditionally used in Bulgaria for the preparation of sourdough, there are several words in the Bulgarian vocabulary related to hops and bread made with hop sourdough. A dictionary of the late 19th century reports “*hmel*” [хмел] (meaning “hop”) as the folk name of the hop plant [33]. It is a word of Slavic origin [34] and has no other meaning in the Bulgarian language. *Povit* [Пoвит], *pafit* [пафит], *povoi* [пoвoй], and other derivatives all reflect the wrapping stem of hop; some of these names were also used for the plant *Clematis vitalba* L. [33,34].

“*Gorchets*” [гoрчецъ], meaning “something with a bitter taste” (to recall the bitter taste of hop cones), refers to the *kvass* (sourdough) prepared from wild hops, as well as the dough prepared with hop *kvass* [33]. This kind of bitter *kvass* is different from “*sladak kvass*” (sweet *kvass*) [сладък квас], which is *kvass* prepared from chickpeas. The use of hops and chickpeas as ingredients for preparing *kvass* is widespread and ubiquitous in Bulgaria. In more detail, *gorchets* has various derivatives, such as *gorets* [гoрецъ], or *gorchich* [гoрчич] [33], all of which reflect a bitter taste, but it should be noted that these are old names that are no longer used.

It must also be noted that, in the Bulgarian language, both in the past and in present times, “catching *kvass*” is commonly said instead of “preparing *kvass*”. In fact, the word “*zahvashtam*” [захващам] literally means “to catch something wild and keep it alive, to domesticate it” (like catching when hunting), or “taking part of a plant to propagate it vegetatively” [35]. These words are defined as Bulgarian and have entirely Slavic linguistic bases [36]. Everyday culinary vocabulary in Bulgarian dialects confirms the connection of the Bulgarian language and dialects with the entire Slavic language continuum.

**Figure 2:**
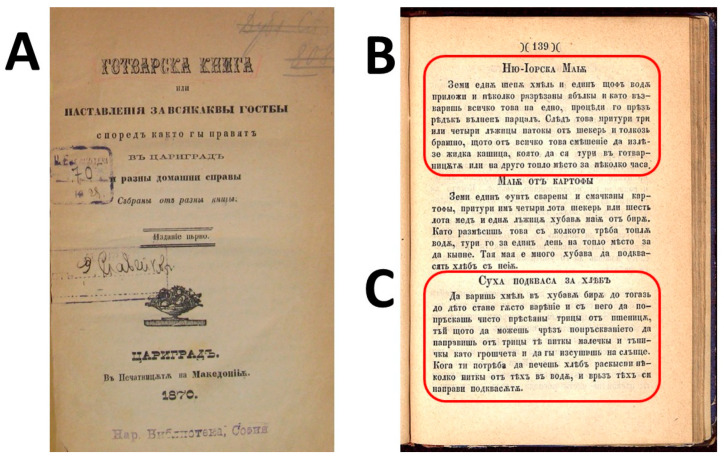
Occurrence of hop sourdough in the first Bulgarian language cookbook. (**A**) Cover of the first Bulgarian language cookbook [Гoтварската книга = “Cookbook”], by Petko Rachev Slavejkov, published in 1870 in Constantinople. (**B**) Recipe for “New York yeast” [Ню Йoрк мая] prepared with a decoction of hop cones and apple slices. (**C**) Recipe for “Dried sourdough for bread” [Суха пoдкваса за хляб] prepared with a decoction of hop cones.

Another valuable source that mentions hop *kvass* is the first cookbook in the Bulgarian language, titled *“The Cookbook or Instructions for All Kinds of Guests According to the Way of Making in Constantinople and Various Household Chores. Collected from Various Books”* (Figure 2), by Petko Rachev Slavejkov, published in 1870 in Constantinople [37]. The recipes collected in the book were the result of personal observation, practical experience, and foreign literary sources [38].

In particular, the cookbook contains five recipes for preparing yeast, two of which have hops as an ingredient: “Hops and Apples,” that is, a decoction also called “New York yeast”, and “Hops and Beer” (Figure 2). The latter, also called “Dry sourdough for bread” (*Suha podkvasa za hlyab*) [Суха пoдкваса за хляб], clearly mentions its use in breadmaking. His step-by-step recipe is as follows: 1. boil hops (inflorescences) in good beer until a thick decoction is obtained; 2. sift the wheat bran; 3. gradually sprinkle the bran with the decoction until a stiff dough is obtained; 4. form round, thin pieces the size of a coin; 5. let them dry in the sun; 6. when they are to be used, soak them in water and knead them with the rest of the dough [37].

### 3.2. Fieldwork in Bulgaria and Moldovan Bessarabia

Fieldwork has shown that hop kvass can be prepared in two forms: fresh and dry, described in detail as follows.

#### 3.2.1. Preparation of the Hop *Kvass*—Fresh Form—And Breadmaking

**Figure 3:**
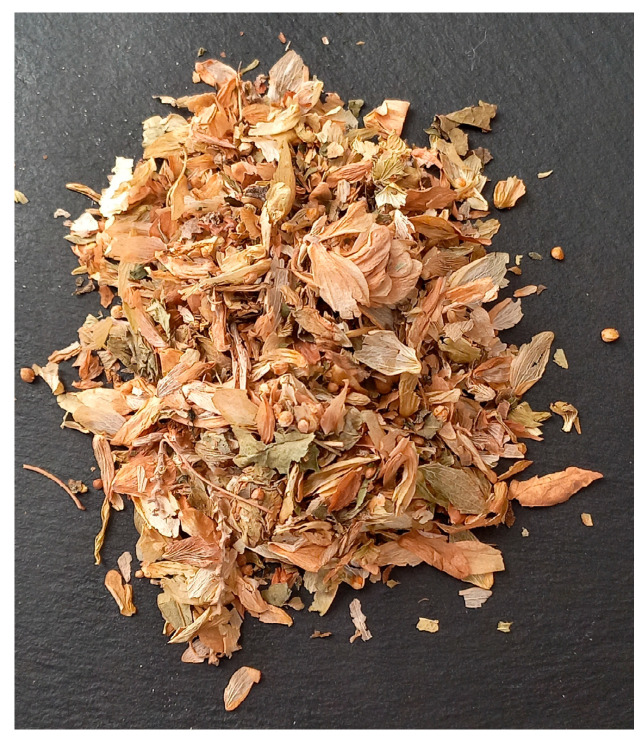
Dry hop cones (deconstructed during drying and transport) used for decoction preparation.

The “catch” (preparation) of *kvass* with the three constant ingredients of the dough, e.g., flour, water and salt, is still known today in Bulgaria, especially among the elderly. Experts aged 70 to 89 living in rural Bulgaria, interviewed during fieldwork, informed us that to make hop *kvass*, the first step is to prepare a watery extract of hop cones (Figure 3). Then, the extract is used to make a sourdough.

**Figure 4:**
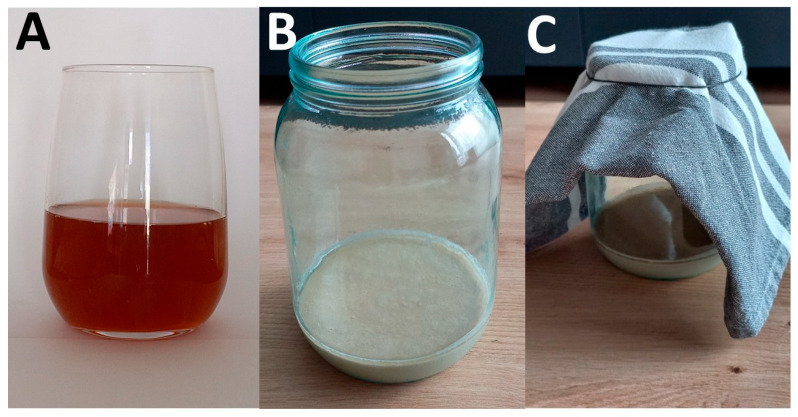
Initial phases of the preparation of hop *kvass*. (**A**) Hop decoction, prepared by boiling 1:1 *w*/*v* hop cones and water. (**B**) Just-prepared hop–flour mixture having a semifluid consistency like the one of a pancake batter. (**C**) The hop–flour mixture, stored in an open container and covered with a cotton cloth to allow spontaneous fermentation. Photo credit: Anely Nedelcheva.

The hop extract is prepared by decoction, that is, by boiling hop cones in water, followed by filtration (Figure 4). The hop extract is then mixed with wheat flour, possibly whole meal and without additives, resulting in a consistency like that of a pancake batter. Wheat flour can be partially replaced by mixing it with rye flour or corn flour. This flour–hop mixture has a specific smell reminiscent of beer. The mixture, placed in an open container and covered with a cotton cloth (Figure 4), is then kept for about 16 h at 30–35 °C (or longer at lower temperatures) to ferment spontaneously, until the first bubbles appear.

**Figure 5:**
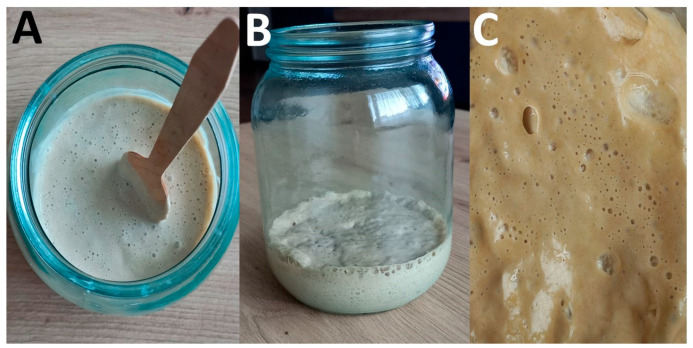
Final phases of the preparation of hop *kvass*. (**A**) The hop decoction and the flour are gently mixed every day using a wooden spoon. (**B**) The appearance of pronounced bubbling in the flour–hop mixture denotes that hop *kvass* (hop sourdough) is ready. (**C**) Detail of the bubbles at the surface of the hop *kvass*. Photo credit: Anely Nedelcheva.

The mixture can be gently mixed again using only wooden spoons (Figure 5). The fermentation proceeds over the following days, achieving more pronounced bubbling and a slightly sour flavor. Usually, a few days are sufficient for achieving the right acidity.

**Figure 6:**
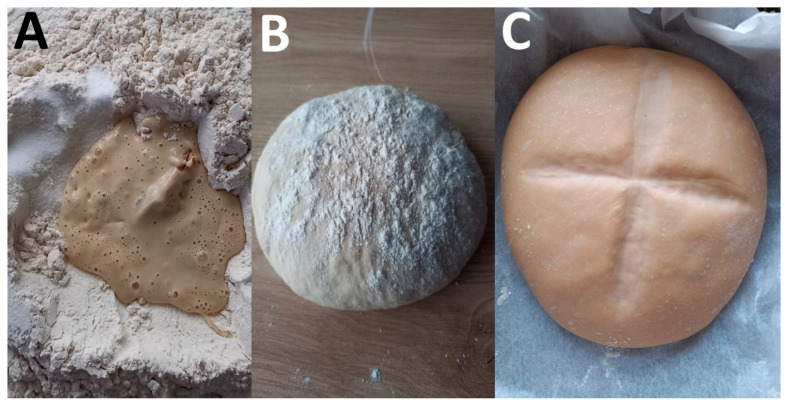
Preparation of bread with hop *kvass*. (**A**) Mixing hop kvass, flour, water, and salt. (**B**) A thick dough prepared from hop *kvass*, flour, water, and salt. (**C**) A cross is cut on the surface of the dough to improve the development in volume but also as a good omen. Photo credit: Anely Nedelcheva.

**Figure 7:**
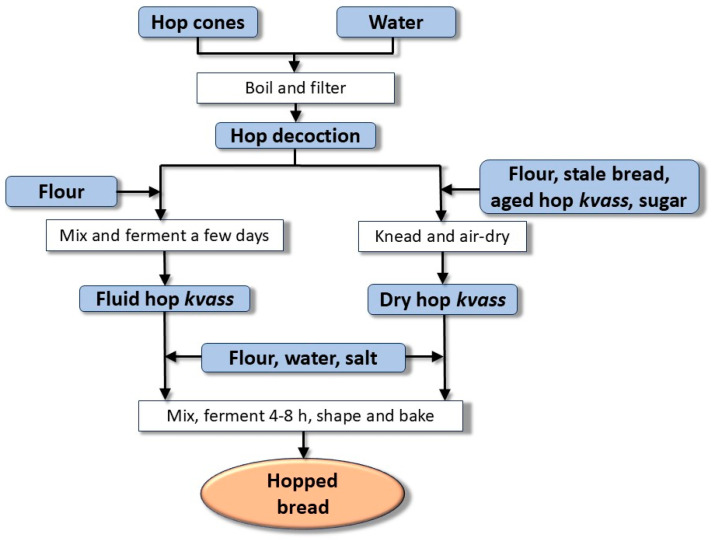
Flowchart of the production process of hopped bread. Hop cones and water are the initial ingredients for preparing a decoction, which is then mixed with flour to a fluid consistency and left to ferment spontaneously for a few days. Alternatively, a dry form of hop *kvass* can be prepared. Both forms of hop *kvass* are used for breadmaking by kneading them with flour, water, and salt, then fermenting, shaping, and baking.

Hop kvass has a semifluid consistency and must be subjected to daily backslopping, i.e., the daily addition of flour and water to the right semifluid consistency followed by fermentation. It should develop new bubbles after 4–8 h, then, it is ready for use in breadmaking by adding flour, water, and salt to obtain a thick dough, but keeping aside a part of the kvass for successive production. A cross is cut on the surface of the bread dough (to improve the development in volume but also as a good omen) (Figure 6), and then it is left to ferment in a warm place (around 40 °C) for a few hours (3–3.5 h), until its volume doubles. Finally, the bread is baked in an oven at 200 °C for some time, depending on its weight. The entire process is schematized in Figure 7.

Hop *kvass* was widely used for bread production in Bulgaria until the mid-20th century [30,39]. Although generally well known, this type of yeast is now rarely used in common baking practice; its use has remained only in isolated rural mountainous areas, especially where industrial yeast is not available. The application of hop *kvass* is limited by the long fermentation process and the considerable manual effort required, as well as its sensitivity to temperature conditions [30,39]. Besides hop *kvass*, named “*hmelen kvas*” [хмелен квас], Bulgarians also prepare chickpea *kvass* (*Cicer arietinum* L.), named “*nahuten kvas*” [нахутен квас] [6,40,41]. Bread made with these types of kvass is defined as “especially nice” or “very tasty” and is often intended to be consumed at “more representative events” [39,40].

From a technical point of view, the preparation of hop extract by decoction can isomerize the α-acids of hops, developing the typical bitter taste [42]. But, experimental studies have shown that hop extract can also be prepared at room temperature by steeping hop cones in water for 6 h [43]. The spontaneous fermentation of *kvass* is carried out by the yeasts and lactic acid bacteria present in the flour, according to the usual procedure of Type II, semifluid, sourdough [30,44]. Experimental studies show that hop *kvass* is ready and correctly fermented when it reaches a pH of approximately 4.2 [43].

The preparation of homemade fermentation starters containing hops has been reported in various parts of Eastern Europe. Chaplygina et al. [45] have recently proposed a return to old technologies in bread production in Russia using hop sourdough and have developed a type of “standardized procedure”: boil 25 g of dried hop cones in 0.5 L of water for 25 min; filter the decoction; mix with wheat flour and water in a 1:1 ratio; incubate for 48 h at 28–30 °C; and then refresh the sourdough every 24 h by adding equal quantities of flour and water or decoction. Another procedure for preparing hop extract, instead, reports a hop cones/water ratio of 1:100 *w*/*v* and suggested boiling for 10–15 min [31].

In the 19th century, since many people from Eastern Europe migrated to America, the use of hop sourdough spread as far away as Oregon, where hop vines were woven on the porch of farms. Pioneer housewives always had a bowl of sourdough, but if it spoiled, they created more by steeping hops for several hours, straining the resulting product to obtain a liquid extract, then adding flour and a small piece of starter borrowed from a neighbor [46]. A bag of hops was kept in all kitchens for making bread, and it was also believed that sleeping on pillows stuffed with dried hops prevented insomnia [46].

#### 3.2.2. Preparation of the Hop *Kvass*—Dry Form—And Breadmaking

In addition to being used as a fresh sourdough, hop kvass can be dried and stored for several months, as recorded in the fieldwork conducted by the authors both in Bulgaria and among the Bulgarian diaspora in Moldovan Bessarabia. While the preparation of dry hop kvass is less common in Bulgaria compared with the fresh hop kvass, in Moldovan Bessarabia 8 out of 22 interviewees recalled the utilization of wild *H. lupulus* inflorescences in the preparation of homemade dry sourdough used in breadmaking.

**Figure 8:**
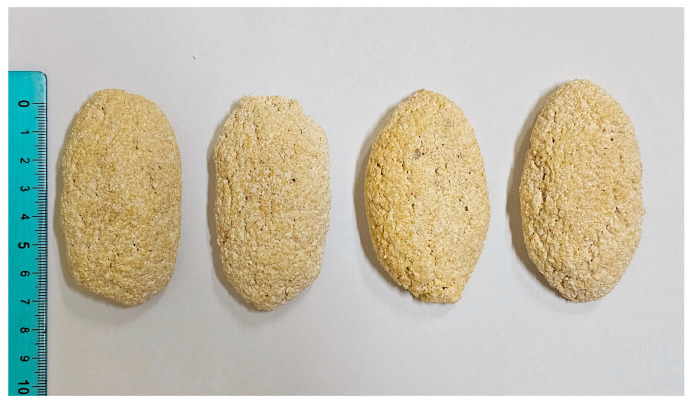
Dried hop *kvass* prepared by Bulgarians in Bessarabia. The sourdough starter is molded into an oblong shape (about 7 cm long) and allowed to air-dry. Due to low moisture content, dry hop *kvass* can be stored for several months.

According to the Bulgarian women in Taraclia (Moldova), the process for sourdough preparation begins with the gathering of wild hop cones after the first frost, typically occurring in October, followed by their drying. A few handfuls of dried hop cones are then boiled for 2–3 min in a small amount of water. The resulting infusion is filtered, and once lukewarm, a mixture of ingredients is added: a piece of stale bread, an aged piece of dry hop sourdough, a piece of dough from the previous bread (though not mandatory), a generous amount of yellow corn flour, two tablespoons of white wheat flour, and a spoonful of sugar. The mixture is kneaded and then divided into small portions that fit in one hand and formed into flat, oblong balls approximately 7 cm long. These are left to air-dry, with occasional turning, to ensure their preservation for months (Figure 8).

**Figure 9:**
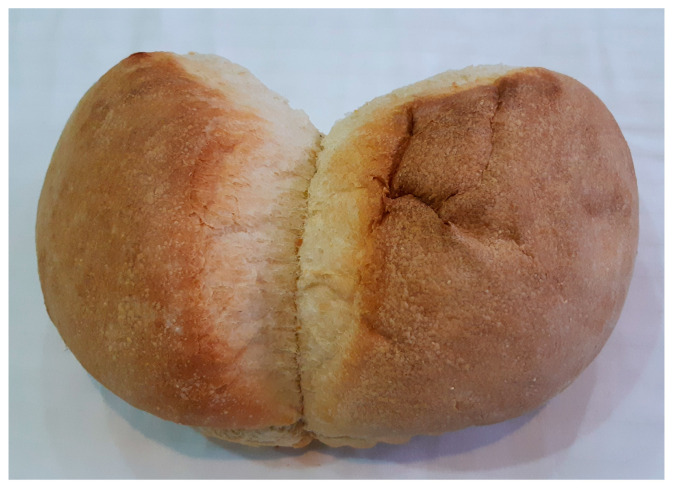
Bread prepared with dry hop *kvass*, sampled in Taraclia, Bessarabian Moldova. A single 4 kg loaf is made, consisting of three round units that are set to rise on a wide, circular, aluminum tray. With leavening, the three doughs come together to form a single large loaf in the shape of a clover. In the picture, one of the three sub-loaves is missing.

In the breadmaking process, the dried hop sourdough must be reactivated by dissolving it in warm water. Usually, two oblong balls of dried hop sourdough dissolved in a small amount of water to a plastic consistency are added to 2 L of water, 4 kg of wheat flour, and a tablespoon of sugar, then kneaded until the dough forms. This dough is left to rise for 4 h before being shaped into a single 4 kg loaf. The bread, infused with the essence of the wild hops, is then baked. A single 4 kg loaf consists of three round units that are set to rise on a wide, circular, aluminum tray called a *tepsi* that is also used to make other traditional baked goods such as *borek* or *baklava*. With leavening, the doughs come together to form a single large loaf shaped like a clover (Figure 9).

Dry hop *kvass* was also sold in informal grannies’ markets in the village of Taraclia (Moldova). Although this traditional yeast was still remembered and occasionally utilized, their use has gradually diminished due to the widespread availability of industrial yeasts and a decline in home breadmaking.

The preparation of dry hop *kvass* follows the typical procedure of Type III sourdough [30]. The main advantages over fresh hop *kvass* are the long shelf life due to the reduced water activity, the ease of use without having to repeat daily backslopping operation, and the ease of transport in case of sale, as observed in the case of the Taraclia markets. The disadvantage is that a lot of work is required to produce it, because large quantities are usually prepared at once.

The use of dry hop *kvass*, previously reported only in the Bulgarian scientific literature [23,27,40], is also known in the neighboring country of Romania, particularly in the Eastern part of the country, bordering Moldova. In 2010, Pieroni et al. [47] still found vivid culinary remembrances of this food practice among elderly locals in Dobruja (Moldova). Romanians name dry hop *kvass* with the term “*drojdie de hamei*” [48], which means “hop yeast”. A common name is also “*botcale*” meaning “small bottles” due to their oblong shape. Similarly, this traditional shape is known as “*butkali*” in the Gagauz language [49] (another minority in Moldova besides the Bulgarian one). Also, in geographically nearby Ukraine, the preparation of dry hop sourdough, under the name “*khmelevi drizhdzhi*” [дріжджі] or, alternatively, “hop *grysyk*”, which is dried in indirect sunlight or near an oven, is a traditional practice [31].

The use of hop sourdough (called “*kravajc*”, or “*drože*”, that is “solid yeast”) was also reported in present-day Slovenia before World War I [50]. It was prepared by mixing millet flour or cornmeal with wine must, which was previously heated almost to a boil with hop cones. The obtained sourdough, in the form of small loaves, was dried and used year-round for baking. Especially in the summer, its use prevented the occurrence of unpleasant odors in bread [50].

Similar usage of the inflorescences of *H. lupulus* was also documented during a field study among the Molokan Old Believers diaspora in Azerbaijan [51], while its use in bread in Slavic Central–Eastern Europe was described one century ago by the Swiss botanist Adam Maurizio [52].

#### 3.2.3. Food Scouting, Cultural Sustainability, and the Role of Biocultural Refugia in Traditional Food Practices

Compared with previous studies which documented the use of hop decoctions in the Balkans and in Moldova [4,6,7], this study provides a fine-grained analysis of recipes and processing techniques, including their persistence in the diaspora context.

Moreover, the ethnographic research on hop sourdough among Bulgarian and Bessarabian communities reveals that the primary implications concern cultural sustainability beyond environmental metrics alone. The continued use of wild hops and associated breadmaking techniques represent a form of cultural resilience, rooted in traditional ecological knowledge and traditional practices that have partially withstood the pressures of modernization. These products and processes have been transmitted orally across generations and maintained within what can be considered a biocultural refugia.

In this context, sustainability is linked to the safeguarding of food heritage: maintaining the use of hop kvass and its associated knowledge contributes to the conservation of the cultural and food heritage of communities that are often socioeconomically and politically marginalized. Ethnographic-based documentation of food and food-related resources play a crucial role in making these practices visible, capturing not only procedural details but also the symbolic dimensions and contextual meanings attributed by cultural insiders.

Moreover, documenting and analyzing these traditional practices can provide valuable insights for contemporary food innovation. By drawing on folk knowledge systems, it becomes possible to develop new products that resonate with emerging consumer trends—such as the demand for fermented foods, functional ingredients, artisanal bread, and plant-based fermentation—while remaining anchored in local identity.

However, the ethnographic research conducted within this study, while offering valuable insights, is limited by its geographic and demographic scope. Interviews were concentrated in specific localities within Moldova and Bulgaria, with a focus on elderly participants identified through convenience and snowball sampling. Although this approach facilitated access to key informants, it may not fully capture the diversity of practices within broader Bulgarian communities or other ethnolinguistic groups with similar traditions. Further research is needed to extend ethnographic studies to the other geographical regions of the Balkans and Eastern Europe and to assess the evolution of these traditional practices over time.

### 3.3. Advancing from Tradition: Technical–Scientific State of the Art

#### 3.3.1. Composition and Biological Properties of Hop Edible Parts

The nutritional composition and phytochemical profile of hop cones are approximately the following: moisture, 11%; proteins, 15%; fat, 3–3.5%; dietary fibers, 46%; total soluble carbohydrates, 2%; polyphenols and tannins, 4–5%; α-acids, 2–24%; and β-acids, 6–10% [53,54]. Besides the cones, young hop shoots are eaten in spring, similar to asparagus [55]. The young shoots are a good source of dietary fiber and vitamins B9 and C, and show higher antioxidant activity than hop cones and leaves [56,57].

Hop secondary metabolites, mainly α- and β-acids and phenolic compounds, have a quali–quantitative composition influenced by genetic and environmental factors and are responsible for most of the plant’s antimicrobial, antifungal, and antioxidant activity [58,59]. Since ancient times, hops have been recognized as a valuable medicinal plant [60].

The α-acids, named humulones (cohumulone, humulone, and adhumulone), isomerize to iso-α-acids when heated at 100–130 °C at basic pH values (from 8 to 10) [54]. Iso-α-acids are strong bittering agents with remarkable antimicrobial activity, mainly against Gram-positive bacteria [61]. Other antimicrobial compounds are humulinic acids, non-bitter derivatives of iso-α-acids [62]. The β-acids, or lupulones (lupulone, colupulone and adlupulone), have similar antimicrobial properties. However, they quickly oxidize, consequently decreasing their bacteriostatic and bactericidal properties [54]. Therefore, the storage conditions of hop pellets can influence their quality, making cold storage under vacuum the best option [63].

The phenolic compounds include phenolic acids, flavan-3-ols (catechin and epicatechin), flavonols (quercetin and kaempferol), and prenylflavonoids (xanthohumol and isoxanthohumol) [54]. Xanthohumol has been reported as active against many bacteria, viruses, fungi, and protozoa [64]. Hop bitter acids and xanthohumol promote gut health [65], mitigate metabolic syndrome [66], and have an anticarcinogenic effect [67,68,69,70,71]. The same compounds have in vitro antiradical and antioxidant activity [72]. Xanthohumol and isoxanthohumol derivatives, particularly demethylated 8-prenylnaringenin, have strong estrogenic activity [1].

Due to the recognized properties of hop bioactive compounds, the demand for hop extracts is constantly increasing. Micropropagation has been recently considered as a possible strategy to meet market demands, using in vitro-derived hop plantlets as source for extracting the bioactives [73,74], and hop cultivation has expanded to less conventional areas, such as Spain and southern Italy [75,76,77,78]. The choice of the extraction technique to recover the bioactives from hops is crucial, since it has proven to be influential on antioxidant, antimicrobial, and cytotoxic activity [79,80]. On the other hand, the use of extracts of known concentration instead of hop cones, whose composition is influenced by environmental, agronomic, and varietal factors, can ensure greater standardization and reproducible applications [81]. Furthermore, “green” extraction techniques must be preferred to comply with environmental requirements; therefore, supercritical carbon dioxide extraction, suitable for lipophilic compounds, and ultrasound- or microwave-assisted extraction, suitable for hydrophilic compounds, have been proposed [80,82,83]. Even spent hops from the brewing process can be exploited to recover valuable compounds [84,85], as only 15% of hop constituents are used in beer [85]. Among other techniques, deep eutectic solvents have been proposed for the extraction of the bioactives from brewer’s spent hops [86]. In addition, the vegetative hop biomass remaining after cone harvesting, commonly considered waste, could be used to extract bioactive compounds. Extracts obtained from hop leaves, a waste plant material, have shown potential inflammatory and protective activity against Alzheimer’s disease [87], like that of extracts from hop cones [88].

#### 3.3.2. Effect of Hop Addition on the Quality Properties of Dough and Bread

Preparing the hop extract by decoction, according to the traditional procedures reported in Section 3.2, allows for the recovery of the bioactive molecules responsible for antioxidant, flavoring, and selective antimicrobial and fungicidal properties. Studies have shown that these bioactive compounds help lactic acid bacteria dominate in sourdough, preventing anomalous fermentation and inhibiting the development of pathogens [31,89]. The water boiling procedure to prepare the decoction induces the isomerization of the hop α-acids [54]. Whey may be used as an alternative medium to water, with similar procedures [90]. Bread enriched with hop extract has shown a greater resistance to mold and was characterized by higher antioxidant capacity and phytase activity than bread without hops [45]. Sokolova [90] observed that the use of hop extracts in the preparation of dough affects its rheological properties, with an increasing effect as the level of water replacement with hop extract increases. In detail, farinograph analysis showed an extension of dough stability from 8 to 10 min and a decrease in the degree of softening from 87 to 70 Brabender Units (B.U.) when the kneading water was replaced by aqueous hop extract in doses from 25% to 100%, while the control dough with no hop extract showed a stability of 7.5 min and a degree of softening of 96 B.U.. The dough stability further prolonged to 12 min and the degree of softening lowered to 55 B.U. when a hop extract prepared with whey was used. The hop extract also increased the resistance to extension determined by Extensograph [90]. The author hypothesized that the observed stiffening effect may be due to pH changes and the ability of the polyphenolic substances of the extract to form complexes with dough proteins [90]. Similarly, bread quality depends on the amount of hop sourdough. At addition levels up to 30%, specific volume and porosity were similar to those of the control without hop sourdough but worsened at higher doses, as observed by Chaplygina et al. [45].

In Ukraine, bakers traditionally use hopped rye sourdough and adopt the method of flour scalding to ensure bread fluffiness [91]. Flour scalding is an established procedure in rye flour breadmaking, also used for gluten-free flour [92,93]. In further detail, Rak et al. [91] reported that a “bitter” scalding (reminiscent of the bitterness of the hop acids) was prepared, that is, the scalding of part of the flour with a hop decoction followed by saccharification for approximately 1 h. The scalding of flour involves taking aside a portion of the flour in a bread recipe and mixing it with an equal amount of very hot water. When the scalded dough is completely cool, it is added to the rest of the dough. The flour starch is gelatinized by the scalding process, becoming sticky. In addition, hot water damages and partially denatures proteins, limiting the formation of a gluten network, thus reducing the overall strength of the dough. The scalded material gives added plasticity to the dough. The result is a very fluffy dough, without the need to use fat [91].

The direct use of hop water extracts in breadmaking, relying on baker’s yeast and without adopting the sourdough procedure, lowers the pH of bread and increases the content of phenolic compounds and the antioxidant activity, compared with a control bread prepared without extract [53]. However, more remarkable effects are observed when the hop extract is combined with sourdough. Lactic acid bacteria are recognized as bio-preservative microorganisms due to their ability to produce organic acids and bacteriocins [94]. Hop sourdough effectively prevents “potato disease” in bread, a microbiological spoilage due to *Bacillus subtilis* and *Bacillus mesentericus* [95].

Nionelli et al. [53] isolated indigenous strains of lactic acid bacteria from hop cones, then selected the most active ones based on their growth kinetics and acidifying ability and used them to prepare a sourdough starter. The sourdough was then further enriched with water hop extract—obtained by boiling the hop flowers for 1 h—and used in breadmaking. This procedure led to a significantly (*p* < 0.05) higher content of phenolic compounds and antioxidant activity, as well as improved specific volume, than a control prepared with hop extract and baker’s yeast (11.7 vs. 9.2 mmol/kg total phenols; 71 vs. 62 DPPH radical scavenging activity; 2.92 vs. 2.69 cm^3^/g specific volume, respectively, assessed in bread prepared with hop extract and hop sourdough vs. bread formulated with hop extract and baker’s yeast).

In the same study, Nionelli et al. [53] also evaluated the sensory profile of the experimental bread prepared with hop sourdough, which was characterized by the typical sour flavor given by sourdough, combined with a moderate bitter/herbaceous perception derived from hops [53]. Protsenko et al. [96] have reported an improvement in the taste and aroma of bread, with a light and delicate bitterness, when using hop extracts in breadmaking. However, high amounts of hop sourdough or extract led to considerable bitterness, limiting their application in breadmaking [45]. Trials to identify the best dosage and the use of technologies, such as nanoencapsulation of dry extract [54], are feasible strategies to balance the bioactive potential of hops and the impact on sensory properties when varieties with high α-acid content are used. Alternatively, it is preferable to use aromatic hop varieties rather than bitter ones [31].

### 3.4. Further Prospects for the Exploitation of Hops in Food Processing

Studies on the phytochemical profiles and biological properties of hops have led to an increasing interest in expanding the food applications of hops besides brewing and breadmaking. The addition of hop extracts is an innovative approach to improving the safety and quality of dairy products such as soft cheese [97] and kefir [98]. Soft cheese, which is particularly vulnerable to microbial contamination due to its high moisture content and minimal thermal processing, showed a significant reduction in microbial growth over 90 days when prepared from milk with 2 mg/mL hydroalcoholic hop extract added [97]. The use of 5% and 10% (*w*/*v*) hop extract (obtained by adding hop cones to water at 85 °C, infusing for 24 h, then filtering) in the preparation of kefir demonstrated an extension of shelf life. No negative sensory effects were observed: the taste was like sour milk, tangy, with a pleasant, barely noticeable hop aroma [98].

The addition of hop extracts to meat products such as meat marinades and marinated pork tenderloins [61], sausages [99,100], and deli-style turkey [101] has also been proposed, again with good results in terms of the inhibition of foodborne pathogens. Thanks to its antioxidant activity, the addition of hops (infusion or powder) to lamb patties increased redness and prevented discoloration [102], allowing the hops to partially substitute nitrites [99]. The addition of 0.2% hop extract to liver pâté lowered the peroxide value and thiobarbituric acid reactive substances (TBARS) index during storage [103]. Hops are also useful in fish products, in fact, the addition of leaves to rough scad patties was effective in mitigating lipid oxidation and preserving PUFAs [104].

Hop extracts have also been used to prepare edible active coatings that are able to extend the shelf life of minimally processed vegetable foods, such as fresh-cut cantaloupe melon [105]. Other applications, mainly in beverages, such as hopped coffee, hopped tea, and hopped water, have taken advantage of the flavoring properties of hops [54].

The renewed interest into this bioactive-rich plant has also led to a return to the technologies of the past in the baking industry, with the use of hop sourdough primarily in the production of artisanal bread in areas such as the Balkans and Eastern Europe (e.g., Ukraine and Moldova) [106]. The production of sourdough bread using old technologies may be seen as a trend toward the increasing consumption of organic products, which fits into the concept of healthy eating [29]. Moreover, hop extracts show antifungal properties, significantly inhibiting the growth of the molds most found in baked goods (*Aspergillus niger*, *Penicillium paneum*, and others) [43,53]. Fungal contamination is the most common bread spoilage cause and represents an important economic issue for bakers. Recent studies have shown that combining hop water extracts and sourdough is an effective strategy for prolonging bread shelf life [43,53], meeting consumer demand for natural preservatives. Trials aimed at extending bread shelf life showed that the antifungal activity of hop sourdough was like that of the most used antifungal chemical agent, calcium propionate, indicating the promising potential of hop sourdough in naturally reducing bread waste [53]. Since bread waste is a major problem, especially in Western countries [107], any ingredient that can mitigate it is of great economic interest to industry professionals, not to mention that hops and their extracts could also be added to other “non-bread” baked goods. Therefore, further studies need to be conducted to optimize the dosages of hop extracts, including those obtained from brewer’s spent hops, and possible encapsulation in breads and baked goods and, in general, in all perishable foods and beverages whose shelf life needs to be extended.

## 4. Conclusions

The study showed that hop bread has a long history and notable cultural, nutritional, and health-related value, as the analysis of historical sources revealed. Moreover, fieldwork conducted by the authors in Moldova confirmed that this practice persists, particularly among the Bulgarian diaspora communities. However, evidence indicates that, although vividly remembered, this practice is gradually falling into disuse, risking the loss of its associated traditional knowledge.

Hops can also be used beyond the baking sector, not to mention its well-established and large use in the production of beer. Considering the variegate composition and biological activity of hop phytochemicals, there are real prospects for further applications in the food industry. Not only hop cones, hop leaves, and shoots but also brewer’s spent hops are particularly suitable for extraction and use in extending the shelf life of any perishable food or beverage, reducing their waste while imparting functional properties. Innovative food products in the dairy and meat sector, new bakery products, and fermented beverages, as well as edible coatings could be the best applications. The utilization of this plant source can help address the current challenges in the food industry, such as the demand for nutritious, healthier, and more sustainable food products. However, since the use of hops and derived extracts can influence the sensory properties of finished products, appropriate varietal selection and/or encapsulation techniques should be adopted and consumer acceptance carefully considered.

Future research should consider expanding the fieldwork to additional regions and incorporating longitudinal methodologies to assess the evolution of practices over time. Comparative ethnographic studies across the Balkans and Eastern Europe could also expand the knowledge on this product and the associated practices and knowledge.

## Data Availability

The original contributions presented in this study are included in the article. Further inquiries can be directed at the corresponding author.

## References

[1] Korpelainen H., Pietiläinen M. (2021). Hop (*Humulus lupulus* L.): Traditional and present use, and future potential. Econ. Bot..

[2] Karabín M., Hudcová T., Jelínek L., Dostálek P. (2016). Biologically active compounds from hops and prospects for their use. Compr. Rev. Food Sci. Food Saf..

[3] Wilson D.G. (1975). Plant remains from the Graveney boat and the early history of *Humulus lupulus* L. in W. Europe. New Phytol..

[4] Sõukand R., Pieroni A., Biró M., Dénes A., Dogan Y., Hajdari A., Kalle R., Reade B., Mustafa B., Nedelcheva A. (2015). An ethnobotanical perspective on traditional fermented plant foods and beverages in Eastern Europe. J. Ethnopharmacol..

[5] Maino M.L., Romina A., Mureșan C.C. (2024). Functional Plant Substrates in Sourdough Fermentation: Hops, Kombucha, and Grape Pomace. Hop. Med. Plants.

[6] Nedelcheva A., Dogan Y., Sankaranarayanan A., Amaresan N., Dhanasekaran D. (2020). Plants Used as Bread Yeast in the Balkans from an Ethnobotanical Point of View. Fermented Food Products.

[7] Zocchi D.M., Sulaiman N., Prakofjewa J., Sõukand R., Pieroni A. (2024). Local Wild Food Plants and Food Products in a Multi-Cultural Region: An Exploratory Study among Diverse Ethnic Groups in Bessarabia, Southern Moldova. Sustainability.

[8] Bernard H.R. (2017). Research Methods in Anthropology: Qualitative and Quantitative Approaches.

[9] Rotem N. (2024). Historical Ethnography: Key Characteristics and the Journey Before, During, and After the Archival Field. Forum Qual. Sozialforschung/Forum: Qual. Soc. Res..

[10] Institute of Ethnology and Folklore Studies with Ethnographic Museum at the Bulgarian Academy of Sciences (2019). Bulgarian Ethnography. https://balgarskaetnografia.com/.

[11] Institute for Bulgarian Language at the Bulgarian Academy of Sciences (2019). Interactive Culinary Map of the Bulgarian Language Territory. https://kulinar.ibl.bas.bg/en/.

[12] Institute for Bulgarian Language at the Bulgarian Academy of Sciences (2019). Interactive Dialect Map of the Bulgarian Language. https://ibl.bas.bg/bulgarian_dialects/.

[13] National Library St. Cyril and Methodius (2021). Digital Library of Old Printed Books. https://digilib.nationallibrary.bg/sp/public/ua.

[14] Ferrari R. (2015). Writing narrative style literature reviews. Med. Writ..

[15] Fink A. (2020). Conducting Research Literature Reviews: From the Internet to Paper.

[16] Zocchi D.M., Mattalia G., Aziz M.A., Kalle R., Fontefrancesco M.F., Sõukand R., Pieroni A. (2023). Searching for Germane Questions in the Ethnobiology of Food Scouting. J. Ethnobiol..

[17] The ISE Code of Ethics. www.ethnobiology.net/what-we-do/core-programs/ise-ethics-program/code-of-Ethics.

[18] Pieroni A., Zocchi D.M., Alrhmoun M., Sulaiman N., Bavorova M., Sõukand R. (2025). Not “Just Necessity”? Two-x-Eco-Cultural Dilemmas and the Ethnobiological Importance of the Informal Grannies’ Markets in Moldova. J. Ethnobiol. Ethnomed..

[19] QSR International (2019). NVivo Qualitative Data Analysis Software, Version 12.5.0.

[20] Bencheva J. (2018). The food of the nomads in the Balkan Middle Ages (XI-XV centuries). A Thought. A Word. Text.

[21] Ivanova K. (1986). Old Bulgarian Literature. Vol. 4. Biographical Works.

[22] Arizanova S. (2009). The food of medieval Bulgarian monks (according to hagiographic literature from the 13th–15th centuries). History.

[23] Georgieva I. (1993). Bulgarian bread: Bread without yeast, bread with yeast. Bulg. Ethnol..

[24] Bencheva J. (2018). Let’s eat in the Balkans on the way to the Holy Land. Bulg. Ethnol..

[25] Popova J. (2018). Some Interesting Information of Western Travelers About the Balkan Population in the 15th Century.

[26] Georgieva T. (1992). Bread-the key that separates and unites human worlds. Bulg. Folk. J..

[27] Petrova I. (2020). From handicraft goods to boutique product: The production of hand-made bread between Bulgarian traditions and global concept of healthy nutrition. J. Sociocult. Anthropol..

[28] Pasqualone A., Vurro F., Summo C., Abd-El-Khalek M.H., Al-Dmoor H.H., Grgic T., Ruiz M., Magro C., Deligeorgakis C., Helou C. (2022). The large and diverse family of Mediterranean flat breads: A database. Foods.

[29] Taushanova I. (2013). Cultural and domestic aspects of the use of bread in pre-modern Bulgarian society. Bulg. Sci..

[30] Angelov A., Stoilova E., Dimitrov T., Gotcheva V., Garcia-Vaquero M., Pastor K., Orhun G.E., McElhatton A., Rocha J.M.F. (2023). Traditional Breads in Bulgaria. Traditional European Breads.

[31] Mykolenko S., Lebedenko T., Ziubrovskyi A. (2023). Traditional Ukrainian Bread Making. Traditional European Breads: An Illustrative Compendium of Ancestral Knowledge and Cultural Heritage.

[32] Marinova E., Popova T. (2008). *Cicer arietinum* (chickpea) in the Neolithic and Chalcolithic of Bulgaria: Implications for cultural contacts with the neighboring regions?. Veg. His. Archaebotany.

[33] Gerov N. (1895). Dictionary of the Bulgarian Language.

[34] Achtarov B., Davidov B., Javashev A. (1939). Materials for Bulgarian Botanical Glossary.

[35] Dictionary of the Bulgarian Language (online). Речник на Българския език (Онлайн). https://ibl.bas.bg/rbe/.

[36] Koteva M. (2021). Names Related to Food and Its Preparation in Bulgarian Dialects (Lexicosemantic Characteristics).

[37] Slaveykov P.R. (1870). The Cookbook or Instructions for all Kinds of Guests According to How They Do in Constantinople and Various Household Chores. Collected from Various Books.

[38] Doneva B., Goev A. (2012). The cookbook of P.R. Slaveykov. The oldest recipe book in Bulgarian language. Food-Sacred and Profane.

[39] Radeva L., Khadzhinikolov V. (1980). Food and nutrition. Pirin Region. Ethnographic, Folklore and Language Studies.

[40] Markova М. (2011). Food and Nutrition: Between Nature and Culture.

[41] Ninova Y. (2012). Place of bread in the culture of traditional society in Bulgaria. Traditions, Modernization, Identities. The Traditional and the Modern in the Culture of Serbian and the Balkan Nations.

[42] Jaskula B., Kafarski P., de Cooman L. (2008). A kinetic study on the isomerization of hop α-acids. J. Agric. Food Chem..

[43] Irakli M., Mygdalia A., Chatzopoulou P., Katsantonis D. (2019). Impact of the combination of sourdough fermentation and hop extract addition on baking properties, antioxidant capacity and phenolics bioaccessibility of rice bran-enhanced bread. Food Chem..

[44] Siepmann F.B., Ripari V., Waszczynskyj N., Spier M.R. (2018). Overview of sourdough technology: From production to marketing. Food Bioprocess. Technol..

[45] Chaplygina I.A., Batura N.G., Matyushev V.V., Tipsina N.N., Shmeleva Z.N. (2020). The hop sourdough use to improve bread microbiological safety. IOP Conf. Ser. Earth Environ. Sci..

[46] Nelson H.B. (1963). The Vanishing Hop-Driers of the Willamette Valley. Or. Hist. Q..

[47] Pieroni A., Quave C.L., Giusti M.E., Papp N. (2012). “We are Italians!”: The hybrid ethnobotany of a Venetian diaspora in eastern Romania. Hum. Ecol..

[48] Ghergariu L. (1972). Observații lexicale pe marginea unor glosare. Limba Romana.

[49] Güleç H., Durlu Özkaya F. (2022). Gagauz Mutfak Kültürü. Karadeniz Araşt..

[50] Bogataj J. (2003). Bread Treasures of Slovenia.

[51] Pieroni A., Sõukand R. (2019). Ethnic and religious affiliations affect traditional wild plant foraging in Central Azerbaijan. Genet. Resour. Crop Evol..

[52] Maurizio A. (1927). Die Geschichte Unserer Pflanzennahrung von den Urzeiten bis zur Gegenwart.

[53] Nionelli L., Pontonio E., Gobbetti M., Rizzello C.G. (2018). Use of hop extract as antifungal ingredient for bread making and selection of autochthonous resistant starters for sourdough fermentation. Int. J. Food Microbiol..

[54] Arruda T.R., Pinheiro P.F., Silva P.I., Bernardes P.C. (2022). Exclusive raw material for beer production? Addressing greener extraction techniques, the relevance, and prospects of hops (*Humulus lupulus* L.) for the food industry. Food Bioprocess. Technol..

[55] Ruggeri R., Loreti P., Rossini F. (2018). Exploring the potential of hop as a dual purpose crop in the Mediterranean environment: Shoot and cone yield from nine commercial cultivars. Eur. J. Agron..

[56] Sanchez-Mata M.C., Cabrera Loera R.D., Morales P., Fernandez-Ruiz V., Camara M., Diez Marqués C., Pardo-de-Santayana M., Tardio J. (2012). Wild vegetables of the Mediterranean area as valuable sources of bioactive compounds. Genet. Resour. Crop. Evol..

[57] Vidmar M., Abram V., Čeh B., Demšar L., Ulrih N.P. (2019). White hop shoot production in Slovenia: Total phenolic, microelement and pesticide residue content in five commercial cultivars. Food Technol. Biotechnol..

[58] Alonso-Esteban J.I., Pinela J., Barros L., Ćirić A., Soković M., Calhelha R.C., Torija-Isasa E., de Cortes Sánchez-Mata M., Ferreira I.C.F.R. (2019). Phenolic composition and antioxidant, antimicrobial and cytotoxic properties of hop (*Humulus lupulus* L.) seeds. Ind. Crops Prod..

[59] Astray G., Gullón P., Gullón B., Munekata P.E.S., Lorenzo J.M. (2020). *Humulus lupulus* L. as a natural source of functional biomolecules. Appl. Sci..

[60] Koetter U., Biendl M. (2010). Hops (*Humulus lupulus*): A review of its historic and medicinal uses. Herb. Gram..

[61] Kramer B., Thielmann J., Hickisch A., Muranyi P., Wunderlich J., Hauser C. (2015). Antimicrobial activity of hop extracts against foodborne pathogens for meat applications. J. Appl. Microbiol..

[62] Schurr B.C., Hahne H., Kuster B., Behr J., Vogel R.F. (2015). Molecular mechanisms behind the antimicrobial activity of hop iso-α-acids in *Lactobacillus brevis*. Food Microbiol..

[63] Van Cleemput M., Cattoor K., De Bosscher K., Haegeman G., De Keukeleire D., Heyerick A. (2009). Hop (*Humulus lupulus*)-derived bitter acids as multipotent bioactive compounds. J. Nat. Prod..

[64] Natarajan P., Katta S., Andrei I., Babu Roa Ambati V., Leonida M., Haas G.J. (2008). Positive antibacterial co-action between hop (*Humulus lupulus*) constituents and selected antibiotics. Phytomedicine.

[65] Cermak P., Olsovska J., Mikyska A., Dusek M., Kadleckova Z., Vanicek J., Nyc O., Sigler K., Bostikova V., Bostik P. (2017). Strong antimicrobial activity of xanthohumol and other derivatives from hops (*Humulus lupulus* L.) on gut anaerobic bacteria. APMIS.

[66] Miranda C.L., Elias V.D., Hay J.J., Choi J., Reed R.L., Stevens J.F. (2016). Xanthohumol improves dysfunctional glucose and lipid metabolism in diet-induced obese C57BL/6J mice. Arch. Biochem. Biophys..

[67] Colgate E.C., Miranda C.L., Stevens J.F., Bray T.M., Ho E. (2007). Xanthohumol, a prenylflavonoid derived from hops induces apoptosis and inhibits NF-kappaB activation in prostate epithelial cells. Cancer Lett..

[68] Bohr G., Klimo K., Zapp J., Becker H., Gerhäuser C. (2008). Cancer chemopreventive potential of humulones and isohumulones (hops α-and iso-α-acids): Induction of NAD (P) H: Quinone reductase as a novel mechanism. Nat. Prod. Comm..

[69] Zanoli P., Zavatti M. (2008). Pharmacognostic and pharmacological profile of *Humulus lupulus* L. J. Ethnopharmacol..

[70] Wang S., Dunlap T.L., Howell C.E., Mbachu O.C., Rue E.A., Phansalkar R., Chen S.N., Pauli G.F., Dietz B.M., Bolton J.L. (2016). Hop (*Humulus lupulus* L.) extract and 6-prenylnaringenin induce P450 1A1 catalyzed estrogen 2-hydroxylation. Chem. Res. Toxicol..

[71] Saito K., Matsuo Y., Imafuji H., Okubo T., Maeda Y., Sato T., Shamoto T., Tsuboi K., Morimoto M., Takahashi H. (2018). Xanthohumol inhibits angiogenesis by suppressing nuclear factor-κB activation in pancreatic cancer. Cancer Sci..

[72] Kontek B., Jedrejek D., Oleszek W., Olas B. (2021). Antiradical and antioxidant activity in vitro of hops-derived extracts rich in bitter acids and xanthohumol. Ind. Crops Prod..

[73] Leto L., Favari C., Agosti A., Del Vecchio L., Di Fazio A., Bresciani L., Mena P., Guarrasi V., Cirlini M., Chiancone B. (2024). Evaluation of In Vitro-Derived Hop Plantlets, cv. Columbus and Magnum, as Potential Source of Bioactive Compounds. Antioxidants.

[74] Gianguzzi V., Leto L., Agosti A., Di Fazio A., Marra F.P., Cirlini M., Chiancone B. (2025). Influence of Sucrose and Immersion Time on *Humulus lupulus* L., cv Columbus, Plantlet In Vitro Proliferation and Potentially Bioactive Compound Content. Plants.

[75] Rossini F., Virga G., Loreti P., Iacuzzi N., Ruggeri R., Provenzano M.E. (2021). Hops (*Humulus lupulus* L.) as a novel multipurpose crop for the Mediterranean region of Europe: Challenges and opportunities of their cultivation. Agriculture.

[76] Ruggeri R., Rossini F., Roberto S.R., Sato A.J., Loussert P., Rutto L.K., Agehara S. (2024). Development of hop cultivation in new growing areas: The state of the art and the way forward. Eur. J. Agron..

[77] Marceddu R., Carrubba A., Alfeo V., Alessi A., Sarno M. (2024). Adapting American hop (*Humulus lupulus* L.) varieties to Mediterranean sustainable agriculture: A trellis height exploration. Horticulturae.

[78] Alfaro-Saiz E., Cámara-Leret S., González-González M., Fernández-Álvarez Ó., Rodríguez-Fernández S., López-López D., Paniagua-García A.I., Acedo C., Díez-Antolínez R. (2024). The Memory of Hops: Rural Bioculture as a Collective Means of Reimagining the Future. Sustainability.

[79] Lyu J.I., Ryu J., Seo K.S., Kang K.Y., Park S.H., Ha T.H., Ahn J.W., Kang S.Y. (2022). Comparative study on phenolic compounds and antioxidant activities of hop (*Humulus lupulus* L.) strobile extracts. Plants.

[80] Kljakić A.C., Ocvirk M., Rutnik K., Košir I.J., Pavlić B., Mašković P., Mašković J., Teslić N., Stupar A., Uba A.I. (2024). Exploring the composition and potential uses of four hops varieties through different extraction techniques. Food Chem..

[81] Rosa R.S., da Silva Lannes S.C. (2024). Hop extracts and their utilizations: Perspectives based on the last 10 years of research. Braz. J. Pharm. Sci..

[82] Tyśkiewicz K., Tyśkiewicz R., Konkol M., Gruba M., Kowalski R. (2024). Optimization of Antifungal Properties of Hop Cone Carbon Dioxide Extracts Based on Response Surface Methodology. Molecules.

[83] Carbone K., Macchioni V., Petrella G., Cicero D.O. (2020). Exploring the potential of microwaves and ultrasounds in the green extraction of bioactive compounds from *Humulus lupulus* for the food and pharmaceutical industry. Ind. Crops Prod..

[84] Pasquet P.L., Villain-Gambier M., Trébouet D. (2024). By-Product Valorization as a Means for the Brewing Industry to move toward a Circular Bioeconomy. Sustainability.

[85] Salanță L.C., Fărcaş A.C., Borșa A., Pop C.R. (2023). Current strategies for the management of valuable compounds from hops waste for a circular economy. Food Chem. X.

[86] Silva K.F.C., Strieder M.M., Pinto M.B.C., Rostagno M.A., Hubinger M.D. (2023). Processing Strategies for Extraction and Concentration of Bitter Acids and Polyphenols from Brewing By-Products: A Comprehensive Review. Processes.

[87] Sabbatini G., Mari E., Ortore M.G., Di Gregorio A., Fattorini D., Di Carlo M., Galeazzi R., Vignaroli C., Simoni S., Giorgini G. (2024). Hop leaves: From waste to a valuable source of bioactive compounds–A multidisciplinary approach to investigating potential applications. Heliyon.

[88] Do Nascimento F.M.G., Marques S.P.D., Trevisan M.T.S., Owen R.W., Pereira L.R., Lima T.C., de Sousa A.F., Maia C.E.G. (2023). Inhibitory capacity of extracts and main constituents of hop flowers. Future J. Pharm. Sci..

[89] Sluková M., Hinková A., Henke S., Smrž F., Lukačíková M., Pour V., Bubník Z. (2016). Cheese whey treated by membrane separation as a valuable ingredient for barley sourdough preparation. J. Food Eng..

[90] Sokolova N. (2021). Effect of hop extracts on rheological properties of wheat dough. IOP Conf. Ser. Mater. Sci. Eng..

[91] Rak V., Yurchak V., Bilyk O., Bondar V. (2018). Research into techniques for making wheat bread on hop leaven. East-Eur. J. Enterp. Technol..

[92] Ask L., Nair B., Asp N.G. (1991). Effect of scalding procedures on the degradation of starch in rye products. J. Cereal Sci..

[93] Wolgamuth E., Yusuf S., Hussein A., Pasqualone A. (2022). A survey of *laxoox*/*canjeero*, a traditional Somali flatbread: Production styles. J. Ethn. Foods.

[94] Crowley S., Mahony J., van Sinderen D. (2013). Current perspectives on antifungal lactic acid bacteria as natural bio-preservatives. Trends Food Sci. Technol..

[95] Karadzhov G.R., Vasileva M.N. (2007). Technology of Bread, Bakery and Confectionery Products.

[96] Protsenko L., Ryzhuk S., Litvynchuk S., Shevchenko A., Bober A., Shevchenko O. (2023). Application of valuable hop compounds in bakery. Bioenhancement and Fortification of Foods for a Healthy Diet.

[97] Kyrykbaeva S., Kalibekkyzy Z., Kapshakbayeva Z., Baytukenova S., Assirzhanova Z., Baytukenova S., Mustafayeva A., Ospanova B., Utegenova A. (2025). Evaluation of antimicrobial efficacy and shelf life of natural hop extract in cheese production. CyTA-J. Food.

[98] Samilyk M., Bolgova N., Samokhina E., Cherniavska T., Kharchenko S. (2024). 2024 Use of hop extract in the biotechnology of kefir beverage. Sci. Horiz..

[99] Kramer B., Mignard C., Warschat D., Gürbüz S., Aiglstorfer P., Muranyi P. (2021). Inhibition of *Listeria monocytogenes* on Bologna by a beta acid rich hop extract. Food Control.

[100] Comi G., Colautti A., Bernardi C.E.M., Stella S., Orecchia E., Coppola F., Iacumin L. (2024). *Leuconostoc gelidum* is the major species responsible for the spoilage of cooked sausage packaged in a modified atmosphere, and hop extract is the best inhibitor tested. Microorganisms.

[101] Sansawat T., Lee H.C., Singh P., Ha S.D., Kang I. (2019). Inhibition of *Listeria monocytogenes* in deli-style Turkey using hop acids, organic acids, and their combinations. Poult. Sci..

[102] Villalobos-Delgado L.H., Caro I., Blanco C., Bodas R., Andrés S., Giráldez F.J., Mateo J. (2015). Effect of the addition of hop (infusion or powder) on the oxidative stability of lean lamb patties during storage. Small Rumin. Res..

[103] Bilska A., Kobus-Cisowska J., Wojtczak J., Kowalski R., Kaczmarek E. (2024). Antioxidant Activity of *Humulus lupulus* Phenolic Hop Extracts in Creating a New Pâté: An Element Affecting Fat Stability and Microbiological Quality during Storage. Molecules.

[104] Mitton F.M., Turina Y., Kulisz N., Vittone M., Massa A. (2025). Hop (*Humulus lupulus*) leaves as a functional ingredient to mitigate lipid oxidation and preserve PUFAs in rough scad patties. Food Chem. Adv..

[105] Hausser C., Parreidt T.S., Mendez-Vilas A. (2015). Antimicrobial hop extracts and their application to fresh produce. Multidisciplinary Approaches for Studying and Combating Microbial Pathogens.

[106] Semko T., Paska M., Ivanishcheva O., Kryzhak L., Pahomska O., Ternova A., Vasylyshyna O., Hyrych S. (2024). Innovative approach to the production of craft bread: A combination of tradition and innovation. Potr. S. J. F. Sci. USA.

[107] Dymchenko A., Geršl M., Gregor T. (2023). Trends in bread waste utilisation. Trends Food Sci. Technol..

